# (*E*)-3-[4-(Dodec­yloxy)phen­yl]-1-(2-hydroxy­phen­yl)prop-2-en-1-one

**DOI:** 10.1107/S1600536809014925

**Published:** 2009-04-25

**Authors:** Ibrahim Abdul Razak, Hoong-Kun Fun, Zainab Ngaini, Siti Muhaini Haris Fadzillah, Hasnain Hussain

**Affiliations:** a X-ray Crystallography Unit, School of Physics, Universiti Sains Malaysia, 11800 USM, Penang, Malaysia; b Department of Chemistry, Faculty of Resource Science and Technology, Universiti Malaysia Sarawak, 94300 Kota Samarahan, Sarawak, Malaysia; c Department of Molecular Biology, Faculty of Resource Science and Technology, Universiti Malaysia Sarawak, 94300 Kota Samarahan, Sarawak, Malaysia

## Abstract

In the title compound, C_27_H_36_O_3_, the asymmetric unit consists of two crystallographically independent mol­ecules. The aromatic rings form dihedral angles of 17.1 (2) and 17.6 (2)° in the two molecules. In both mol­ecules, the enone groups adopt an *s*–*cis* conformation and the alkoxyl chains are in *trans* conformations curving out of the zigzag plane. Intra­molecular O—H⋯O hydrogen bonds involving the keto and hydr­oxy groups generate *S*(6) ring motifs. The mol­ecules are stacked alternately in a head-to-tail fashion along the *a* axis and the crystal structure is stabilized by weak C—H⋯π inter­actions. The crystal studied was a non-merohedral twin, the ratio of components being 0.788 (2):0.212 (2).

## Related literature

For general background to the biological activity of chalcone derivatives, see: Bhat *et al.* (2005 ▶); Xue *et al.* (2004 ▶); Satyanarayana *et al.* (2004 ▶); Zhao *et al.* (2005 ▶); Lee *et al.* (2006 ▶). For related structures, see: Ng *et al.* (2006 ▶); Razak *et al.* (2009 ▶); Ngaini, Fadzillah *et al.* (2009 ▶); Ngaini, Rahman *et al.* (2009 ▶). For details of hydrogen-bond motifs, see: Bernstein *et al.* (1995 ▶). For stability of the temperature controller used for the data collection, see: Cosier & Glazer (1986 ▶).
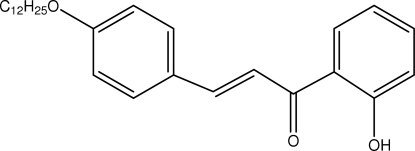


## Experimental

### 

#### Crystal data

C_27_H_36_O_3_
*M* *_r_* = 408.56
Triclinic, 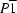

*a* = 7.4953 (6) Å
*b* = 13.4714 (9) Å
*c* = 23.7874 (18) Å
α = 75.116 (4)°
β = 83.876 (5)°
γ = 84.669 (5)°
*V* = 2302.7 (3) Å^3^
*Z* = 4
Mo *K*α radiation
μ = 0.08 mm^−1^
*T* = 100 K
0.55 × 0.13 × 0.06 mm

#### Data collection

Bruker APEXII diffractometer
Absorption correction: multi-scan (*SADABS*; Bruker, 2005 ▶) *T*_min_ = 0.960, *T*_max_ = 0.996
8571 measured reflections
8571 independent reflections
4737 reflections with *I* > 2σ(*I*)

#### Refinement

*R*[*F* ^2^ > 2σ(*F* ^2^)] = 0.069
*wR*(*F* ^2^) = 0.206
*S* = 1.03
8571 reflections
546 parameters
H-atom parameters constrained
Δρ_max_ = 0.29 e Å^−3^
Δρ_min_ = −0.30 e Å^−3^

### 

Data collection: *APEX2* (Bruker, 2005 ▶); cell refinement: *SAINT* (Bruker, 2005 ▶); data reduction: *SAINT* (Bruker, 2005 ▶); program(s) used to solve structure: *SHELXTL* (Sheldrick, 2008 ▶); program(s) used to refine structure: *SHELXTL* (Sheldrick, 2008 ▶); molecular graphics: *SHELXTL* (Sheldrick, 2008 ▶); software used to prepare material for publication: *SHELXTL* (Sheldrick, 2008 ▶).

## Supplementary Material

Crystal structure: contains datablocks global, I. DOI: 10.1107/S1600536809014925/lh2802sup1.cif

Structure factors: contains datablocks I. DOI: 10.1107/S1600536809014925/lh2802Isup2.hkl

Additional supplementary materials:  crystallographic information; 3D view; checkCIF report

## Figures and Tables

**Table 1:** Hydrogen-bond geometry (Å, °)

*D*—H⋯*A*	*D*—H	H⋯*A*	*D*⋯*A*	*D*—H⋯*A*
O1*A*—H1*A*⋯O2*A*	0.82	1.79	2.513 (4)	146
O1*B*—H1*B*⋯O2*B*	0.82	1.81	2.530 (4)	146
C22*B*—H22*C*⋯*Cg*1^i^	0.97	2.77	3.654 (4)	151
C17*B*—H17*D*⋯*Cg*2	0.97	2.82	3.612 (4)	139
C22*A*—H22*B*⋯*Cg*3	0.97	2.93	3.765 (4)	145

Symmetry code: (i) 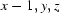. *Cg*1, *Cg*2 and *Cg*3 are the centroids of the C1*A-*-C6*A*, C10*A*–C15*A* and C1*B*–C6*B* rings, respectively.

**Fig. 1.**
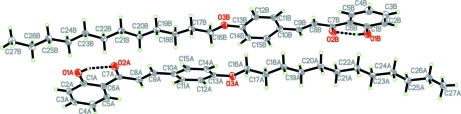
The asymmetric unit of the title compound, showing 50% probability displacement ellipsoids and the atom numbering scheme. Intramolecular interactions were shown as dashed line.

**Fig. 2.**
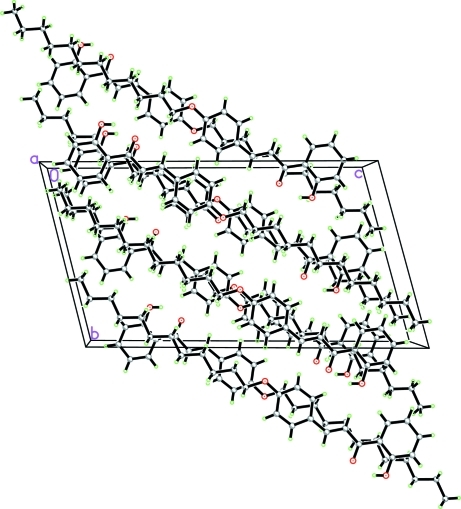
Part of the crystal structure of (I) viewed along the a axis.

## supplementary crystallographic information

### Comment

Chalcone derivatives are of interest because of their biological properties such as anticancer (Bhat *et al.*, 2005), antimalarial (Xue *et al.*, 2004), antiangiogenic and antitumour (Lee *et al.*, 2006), antiplatelet activity (Zhao *et al.*, 2005) and antihyperglycemic activity (Satyanarayana *et al.*, 2004). As part of our studies on their biological properties, we have synthesized the title chalcone derivative, (I). Its antibacterial activities were tested against *E. coli* ATCC 8739 and showed antimicrobial activity. The structure determination of (I) was carried out and is reported in this paper.

The asymmetric unit of (I) consists of two crystallographically independent molecules, A and B (Fig.1). The bond lengths show normal values (Allen *et al.*, 1987). The mean plane through the enone moiety (O2/C7/C8/C9) makes dihedral angles with the two benzene rings with values of 3.4 (2)° (C1—C6) and 16.0 (2)° (C10—C15) in molecule A and 7.8 (2)° (C1—C6) and 15.7 (2)° (C10—C15) in molecule B. The two benzene rings form dihedral angles with each other of 17.1 (2)° and 17.6 (2)° in molecules A and B, respectively.

The enone moieties adopt *s*-*cis* conformation with the O2—C7—C8—C9 torsion angle being 6.5 (5)° for molecule A and 8.8 (5)° for B. In what follows, the distortion of the angles is relative to what is expected in terms of hybridization principles. In molecule A, the slight enlargement of the C5A—C6A—C7A (122.4 (3)°) and C6A—C7A—C8A (121.9 (3)°) angles may be the result of the short H5AA···H8AA (2.14 Å) contact whereas the short H8AA···H11A (2.35 Å) contact may widen the C8A—C9A—C10A (129.0 (3)°) and C9A—C10A—C11A (123.0 (3)°) angles. The short H14A···H16A (2.26 Å) contact may result in the opening of the O3A—C13A—C14A (124.9 (3)°) angle. Likewise, in molecule B, a close interatomic contact between H5BA and H8BA (2.13 Å) may result in the widening of the C5B—C6B—C7B (123.1 (3)°) and C6B—C7B—C8B (121.2 (3)°) angles whereas the opening of C8B—C9B—C10B and C9B—C10B—C11B angles to 128.6 (3)° and 123.0 (3)°, respectively, may be the result of the close H8BA···H11B (2.30 Å) contact. Similar strain induced by a short H14B···H16C (2.26 Å) contact may result in the opening of the O3B—C13B—C14B (125.2 (3)°) angle. These features were also reported in related structures (Ng *et al.*, 2006; Razak *et al.*, 2009; Ngaini, Fadzillah *et al.*, 2009; Ngaini, Rahman *et al.*, 2009).

The conformation throughout the zigzag alkoxyl tails in both molecules is *trans* with the largest deviation from the ideal value being -174.4 (3)° for the C19A—C20A—C21A—C22A torsion angle in molecule A and -173.5 (3)° for the C17B—C18B—C19B—C20B torsion angle in B. Even though the torsion angle C16—O3—C13—C14 in each molecule is -6.8 (5)° for A and -12.2 (5)° for B, the alkoxyl chains curve out of the zigzag plane with the least-squares plane through the chain making dihedral angle with the attached benzene ring of 17.02 (19)° [maximum deviation of -0.302 (4)Å at C21A] and 16.73 (19)° [maximum deviation of -0.256 (4)Å at C21B], for molecules A and B, respectively.

An intramolecular O-H···O interaction involving the keto and hydroxy groups (Table 1) in both molecules generates *S*(6) ring motifs (Bernstein *et al.*, 1995). In the crystal structure, the molecules are stacked alternately along the *a* axis in a head-to-tail manner (Fig. 2). In the absence of conventional hydrogen bonds, the crystal structure is stabilized by weak C—H···π interactions (Table 1).

### Experimental

A mixture of 2-hydroxyacetophenone (2.72 ml, 20 mmol) and 4-dodecyloxybenzaldehyde (5.81 ml, 20 mmol) and KOH (4.04 g, 72 mmol) in 60 ml of methanol was heated at reflux for 10 h. The reaction was cooled to room temperature and acidified with cold diluted HCl (2 N). The resulting precipitate was filtered, washed and dried. After redissolving in hexane, followed by a few days of slow evaporation, crystals were collected.

### Refinement

All H atoms were positioned geometrically and refined using a riding model. The *U*_iso_(H) values were constrained to be 1.5U_eq_(C,O) (methyl H and hydroxyl H atoms) and 1.2U_eq_(C) (other H atoms). The rotating model group was considered for the methyl group. The crystal is a twin with a refined BASF = 0.212 (2).

### Crystal data

**Table:**

C_27_H_36_O_3_	*Z* = 4
*M**_r_* = 408.56	*F*(000) = 888
Triclinic, *P*1	*D*_x_ = 1.179 Mg m^−^^3^
Hall symbol: -P 1	Mo *K*α radiation, λ = 0.71073 Å
*a* = 7.4953 (6) Å	Cell parameters from 3668 reflections
*b* = 13.4714 (9) Å	θ = 2.7–28.0°
*c* = 23.7874 (18) Å	µ = 0.08 mm^−^^1^
α = 75.116 (4)°	*T* = 100 K
β = 83.876 (5)°	Needle, yellow
γ = 84.669 (5)°	0.55 × 0.13 × 0.06 mm
*V* = 2302.7 (3) Å^3^	

### Data collection

**Table:**

Bruker APEXII diffractometer	8571 independent reflections
Radiation source: sealed tube	4737 reflections with *I* > 2σ(*I*)
graphite	*R*_int_ = 0.0000
π and ω scans	θ_max_ = 25.5°, θ_min_ = 0.9°
Absorption correction: multi-scan (*SADABS*; Bruker, 2005)	*h* = −8→9
*T*_min_ = 0.960, *T*_max_ = 0.996	*k* = −16→16
8571 measured reflections	*l* = −28→28

### Refinement

**Table:**

Refinement on *F*^2^	Primary atom site location: structure-invariant direct methods
Least-squares matrix: full	Secondary atom site location: difference Fourier map
*R*[*F*^2^ > 2σ(*F*^2^)] = 0.069	Hydrogen site location: inferred from neighbouring sites
*wR*(*F*^2^) = 0.206	H-atom parameters constrained
*S* = 1.03	*w* = 1/[σ^2^(*F*_o_^2^) + (0.0924*P*)^2^ + 0.763*P*] where *P* = (*F*_o_^2^ + 2*F*_c_^2^)/3
8571 reflections	(Δ/σ)_max_ < 0.001
546 parameters	Δρ_max_ = 0.29 e Å^−^^3^
0 restraints	Δρ_min_ = −0.30 e Å^−^^3^

### Special details

**Table:**

Experimental. The crystal was placed in the cold stream of an Oxford Cryosystems Cobra open-flow nitrogen cryostat (Cosier & Glazer, 1986) operating at 100.0 (1) K.
Geometry. All e.s.d.'s (except the e.s.d. in the dihedral angle between two l.s. planes) are estimated using the full covariance matrix. The cell e.s.d.'s are taken into account individually in the estimation of e.s.d.'s in distances, angles and torsion angles; correlations between e.s.d.'s in cell parameters are only used when they are defined by crystal symmetry. An approximate (isotropic) treatment of cell e.s.d.'s is used for estimating e.s.d.'s involving l.s. planes.
Refinement. Refinement of *F*^2^ against ALL reflections. The weighted *R*-factor *wR* and goodness of fit *S* are based on *F*^2^, conventional *R*-factors *R* are based on *F*, with *F* set to zero for negative *F*^2^. The threshold expression of *F*^2^ > σ(*F*^2^) is used only for calculating *R*-factors(gt) *etc*. and is not relevant to the choice of reflections for refinement. *R*-factors based on *F*^2^ are statistically about twice as large as those based on *F*, and *R*- factors based on ALL data will be even larger.

### Fractional atomic coordinates and isotropic or equivalent isotropic displacement parameters (Å^2^)

**Table:**

	*x*	*y*	*z*	*U*_iso_*/*U*_eq_	
O1A	0.6765 (4)	0.69790 (18)	0.78918 (11)	0.0307 (6)	
H1A	0.6609	0.6985	0.7555	0.046*	
O2A	0.6335 (3)	0.62359 (18)	0.70527 (11)	0.0290 (6)	
O3A	0.6291 (3)	0.19687 (18)	0.50595 (10)	0.0255 (6)	
C1A	0.6917 (5)	0.5994 (3)	0.82144 (16)	0.0249 (8)	
C2A	0.7137 (5)	0.5837 (3)	0.88068 (16)	0.0273 (9)	
H2AA	0.7158	0.6397	0.8966	0.033*	
C3A	0.7324 (5)	0.4852 (3)	0.91544 (16)	0.0294 (9)	
H3AA	0.7456	0.4751	0.9550	0.035*	
C4A	0.7320 (5)	0.4007 (3)	0.89221 (15)	0.0288 (9)	
H4AA	0.7469	0.3344	0.9159	0.035*	
C5A	0.7094 (5)	0.4154 (3)	0.83407 (15)	0.0256 (8)	
H5AA	0.7088	0.3585	0.8187	0.031*	
C6A	0.6871 (4)	0.5145 (3)	0.79750 (15)	0.0210 (8)	
C7A	0.6563 (4)	0.5329 (3)	0.73494 (15)	0.0220 (8)	
C8A	0.6552 (5)	0.4480 (3)	0.70683 (15)	0.0235 (8)	
H8AA	0.6597	0.3802	0.7291	0.028*	
C9A	0.6477 (4)	0.4687 (3)	0.64905 (15)	0.0231 (8)	
H9AA	0.6432	0.5381	0.6297	0.028*	
C10A	0.6455 (4)	0.3974 (3)	0.61220 (15)	0.0214 (8)	
C11A	0.6156 (5)	0.2928 (3)	0.63470 (15)	0.0230 (8)	
H11A	0.5988	0.2660	0.6749	0.028*	
C12A	0.6109 (4)	0.2297 (3)	0.59822 (15)	0.0221 (8)	
H12A	0.5905	0.1606	0.6139	0.027*	
C13A	0.6362 (4)	0.2674 (3)	0.53777 (15)	0.0203 (8)	
C14A	0.6673 (5)	0.3704 (3)	0.51432 (15)	0.0229 (8)	
H14A	0.6862	0.3961	0.4741	0.028*	
C15A	0.6698 (5)	0.4344 (3)	0.55132 (15)	0.0233 (8)	
H15A	0.6882	0.5037	0.5354	0.028*	
C16A	0.6352 (5)	0.2302 (3)	0.44325 (15)	0.0236 (8)	
H16A	0.7410	0.2680	0.4277	0.028*	
H16B	0.5292	0.2746	0.4313	0.028*	
C17A	0.6417 (5)	0.1341 (3)	0.42151 (15)	0.0252 (8)	
H17A	0.5401	0.0951	0.4406	0.030*	
H17B	0.7502	0.0923	0.4334	0.030*	
C18A	0.6386 (5)	0.1513 (3)	0.35641 (15)	0.0233 (8)	
H18A	0.7441	0.1861	0.3368	0.028*	
H18B	0.5331	0.1953	0.3437	0.028*	
C19A	0.6354 (5)	0.0500 (3)	0.33918 (15)	0.0240 (8)	
H19A	0.7360	0.0046	0.3550	0.029*	
H19B	0.5261	0.0178	0.3575	0.029*	
C20A	0.6443 (5)	0.0575 (3)	0.27412 (15)	0.0255 (8)	
H20A	0.7542	0.0885	0.2554	0.031*	
H20B	0.5437	0.1023	0.2578	0.031*	
C21A	0.6396 (5)	−0.0469 (3)	0.26067 (15)	0.0252 (8)	
H21A	0.7325	−0.0935	0.2806	0.030*	
H21B	0.5246	−0.0746	0.2764	0.030*	
C22A	0.6664 (5)	−0.0442 (3)	0.19601 (15)	0.0246 (8)	
H22A	0.7767	−0.0116	0.1794	0.029*	
H22B	0.5678	−0.0025	0.1765	0.029*	
C23A	0.6765 (5)	−0.1509 (3)	0.18409 (15)	0.0251 (8)	
H23A	0.7717	−0.1936	0.2051	0.030*	
H23B	0.5641	−0.1823	0.1992	0.030*	
C24A	0.7112 (5)	−0.1494 (3)	0.11987 (15)	0.0265 (9)	
H24A	0.8193	−0.1139	0.1041	0.032*	
H24B	0.6119	−0.1105	0.0993	0.032*	
C25A	0.7332 (5)	−0.2558 (3)	0.10808 (15)	0.0276 (9)	
H25A	0.8331	−0.2946	0.1284	0.033*	
H25B	0.6254	−0.2915	0.1240	0.033*	
C26A	0.7664 (5)	−0.2538 (3)	0.04410 (15)	0.0288 (9)	
H26A	0.8748	−0.2187	0.0282	0.035*	
H26B	0.6671	−0.2145	0.0237	0.035*	
C27A	0.7868 (6)	−0.3608 (3)	0.03238 (16)	0.0336 (10)	
H27A	0.8210	−0.3548	−0.0084	0.050*	
H27B	0.6745	−0.3925	0.0431	0.050*	
H27C	0.8778	−0.4023	0.0551	0.050*	
O1B	0.1478 (4)	−0.19486 (18)	0.21550 (11)	0.0324 (7)	
H1B	0.1245	−0.1949	0.2500	0.049*	
O2B	0.0945 (3)	−0.11900 (18)	0.30324 (11)	0.0290 (6)	
O3B	0.1502 (3)	0.30711 (17)	0.50023 (10)	0.0251 (6)	
C1B	0.1699 (5)	−0.0980 (3)	0.18370 (16)	0.0248 (8)	
C2B	0.2029 (5)	−0.0831 (3)	0.12353 (17)	0.0296 (9)	
H2BA	0.2081	−0.1390	0.1071	0.036*	
C3B	0.2280 (5)	0.0137 (3)	0.08821 (17)	0.0328 (10)	
H3BA	0.2487	0.0230	0.0480	0.039*	
C4B	0.2224 (5)	0.0976 (3)	0.11249 (16)	0.0310 (9)	
H4BA	0.2395	0.1631	0.0886	0.037*	
C5B	0.1917 (5)	0.0837 (3)	0.17155 (16)	0.0252 (8)	
H5BA	0.1892	0.1402	0.1874	0.030*	
C6B	0.1638 (4)	−0.0137 (3)	0.20895 (16)	0.0224 (8)	
C7B	0.1286 (4)	−0.0313 (3)	0.27275 (15)	0.0224 (8)	
C8B	0.1362 (4)	0.0522 (3)	0.30173 (15)	0.0230 (8)	
H8BA	0.1448	0.1195	0.2794	0.028*	
C9B	0.1308 (4)	0.0320 (3)	0.35977 (15)	0.0217 (8)	
H9BA	0.1227	−0.0367	0.3796	0.026*	
C10B	0.1359 (5)	0.1032 (3)	0.39596 (15)	0.0231 (8)	
C11B	0.1184 (5)	0.2102 (3)	0.37436 (15)	0.0234 (8)	
H11B	0.1037	0.2379	0.3350	0.028*	
C12B	0.1225 (5)	0.2748 (3)	0.41016 (16)	0.0258 (9)	
H12B	0.1100	0.3457	0.3949	0.031*	
C13B	0.1452 (5)	0.2351 (3)	0.46948 (16)	0.0230 (8)	
C14B	0.1622 (5)	0.1297 (3)	0.49237 (16)	0.0249 (8)	
H14B	0.1760	0.1024	0.5319	0.030*	
C15B	0.1585 (5)	0.0651 (3)	0.45546 (16)	0.0259 (9)	
H15B	0.1713	−0.0057	0.4708	0.031*	
C16B	0.1390 (5)	0.2755 (3)	0.56305 (15)	0.0237 (8)	
H16C	0.2351	0.2240	0.5761	0.028*	
H16D	0.0247	0.2466	0.5783	0.028*	
C17B	0.1564 (5)	0.3710 (3)	0.58335 (15)	0.0247 (8)	
H17C	0.0658	0.4232	0.5666	0.030*	
H17D	0.2732	0.3969	0.5684	0.030*	
C18B	0.1366 (5)	0.3554 (3)	0.64911 (15)	0.0245 (8)	
H18C	0.2292	0.3050	0.6663	0.029*	
H18D	0.0206	0.3289	0.6646	0.029*	
C19B	0.1520 (5)	0.4559 (3)	0.66580 (15)	0.0249 (8)	
H19C	0.2613	0.4858	0.6457	0.030*	
H19D	0.0517	0.5031	0.6515	0.030*	
C20B	0.1549 (5)	0.4484 (3)	0.73017 (15)	0.0231 (8)	
H20C	0.2600	0.4055	0.7444	0.028*	
H20D	0.0492	0.4153	0.7510	0.028*	
C21B	0.1587 (5)	0.5525 (3)	0.74315 (15)	0.0248 (8)	
H21C	0.0480	0.5927	0.7321	0.030*	
H21D	0.2572	0.5882	0.7189	0.030*	
C22B	0.1799 (5)	0.5491 (3)	0.80653 (15)	0.0255 (8)	
H22C	0.0780	0.5170	0.8307	0.031*	
H22D	0.2875	0.5063	0.8184	0.031*	
C23B	0.1928 (5)	0.6542 (3)	0.81750 (15)	0.0262 (8)	
H23C	0.0837	0.6963	0.8067	0.031*	
H23D	0.2924	0.6871	0.7924	0.031*	
C24B	0.2195 (5)	0.6512 (3)	0.88035 (15)	0.0262 (8)	
H24C	0.3210	0.6032	0.8925	0.031*	
H24D	0.1137	0.6252	0.9049	0.031*	
C25B	0.2525 (5)	0.7555 (3)	0.89035 (15)	0.0276 (9)	
H25C	0.3601	0.7808	0.8666	0.033*	
H25D	0.1524	0.8041	0.8774	0.033*	
C26B	0.2743 (5)	0.7519 (3)	0.95361 (15)	0.0281 (9)	
H26C	0.3818	0.7090	0.9654	0.034*	
H26D	0.1724	0.7200	0.9779	0.034*	
C27B	0.2879 (6)	0.8576 (3)	0.96430 (17)	0.0361 (10)	
H27D	0.3122	0.8498	1.0041	0.054*	
H27E	0.1764	0.8979	0.9570	0.054*	
H27F	0.3835	0.8915	0.9386	0.054*	

### Atomic displacement parameters (Å^2^)

**Table:**

	*U*^11^	*U*^22^	*U*^33^	*U*^12^	*U*^13^	*U*^23^
O1A	0.0346 (16)	0.0267 (14)	0.0343 (16)	−0.0020 (11)	−0.0117 (13)	−0.0102 (12)
O2A	0.0320 (15)	0.0243 (14)	0.0325 (15)	−0.0041 (11)	−0.0084 (12)	−0.0074 (11)
O3A	0.0314 (15)	0.0256 (14)	0.0220 (14)	−0.0052 (11)	−0.0045 (11)	−0.0081 (11)
C1A	0.0151 (19)	0.027 (2)	0.033 (2)	−0.0025 (15)	−0.0031 (16)	−0.0072 (17)
C2A	0.022 (2)	0.031 (2)	0.034 (2)	−0.0024 (16)	−0.0058 (17)	−0.0160 (17)
C3A	0.025 (2)	0.043 (2)	0.023 (2)	−0.0046 (17)	−0.0076 (17)	−0.0121 (18)
C4A	0.032 (2)	0.029 (2)	0.025 (2)	−0.0052 (17)	−0.0047 (17)	−0.0054 (16)
C5A	0.025 (2)	0.027 (2)	0.027 (2)	−0.0053 (16)	−0.0017 (16)	−0.0102 (16)
C6A	0.0143 (18)	0.027 (2)	0.024 (2)	−0.0053 (15)	−0.0010 (15)	−0.0078 (16)
C7A	0.0136 (18)	0.025 (2)	0.027 (2)	−0.0029 (14)	−0.0030 (15)	−0.0055 (16)
C8A	0.020 (2)	0.024 (2)	0.027 (2)	−0.0006 (15)	−0.0037 (16)	−0.0048 (16)
C9A	0.0148 (19)	0.025 (2)	0.029 (2)	−0.0038 (14)	−0.0057 (16)	−0.0032 (16)
C10A	0.0111 (18)	0.028 (2)	0.028 (2)	−0.0022 (14)	−0.0037 (15)	−0.0102 (16)
C11A	0.0179 (19)	0.029 (2)	0.023 (2)	−0.0024 (15)	−0.0054 (15)	−0.0049 (16)
C12A	0.0167 (19)	0.0210 (19)	0.028 (2)	−0.0016 (14)	−0.0059 (15)	−0.0042 (16)
C13A	0.0132 (18)	0.0224 (19)	0.029 (2)	0.0012 (14)	−0.0062 (15)	−0.0130 (16)
C14A	0.022 (2)	0.027 (2)	0.021 (2)	−0.0014 (15)	−0.0082 (16)	−0.0062 (16)
C15A	0.0210 (19)	0.0197 (19)	0.029 (2)	−0.0052 (15)	−0.0059 (16)	−0.0035 (15)
C16A	0.022 (2)	0.026 (2)	0.025 (2)	−0.0033 (15)	−0.0034 (16)	−0.0098 (16)
C17A	0.022 (2)	0.030 (2)	0.025 (2)	−0.0019 (16)	−0.0032 (16)	−0.0084 (16)
C18A	0.021 (2)	0.025 (2)	0.026 (2)	−0.0010 (15)	−0.0059 (16)	−0.0073 (16)
C19A	0.021 (2)	0.026 (2)	0.025 (2)	−0.0035 (15)	−0.0063 (16)	−0.0045 (16)
C20A	0.020 (2)	0.030 (2)	0.028 (2)	−0.0011 (16)	−0.0066 (16)	−0.0077 (16)
C21A	0.024 (2)	0.0225 (19)	0.031 (2)	−0.0004 (15)	−0.0100 (17)	−0.0057 (16)
C22A	0.022 (2)	0.028 (2)	0.026 (2)	−0.0028 (15)	−0.0058 (16)	−0.0092 (16)
C23A	0.021 (2)	0.027 (2)	0.027 (2)	0.0012 (15)	−0.0087 (16)	−0.0061 (16)
C24A	0.026 (2)	0.029 (2)	0.025 (2)	−0.0020 (16)	−0.0028 (16)	−0.0075 (16)
C25A	0.026 (2)	0.030 (2)	0.028 (2)	−0.0037 (16)	−0.0076 (17)	−0.0067 (16)
C26A	0.032 (2)	0.029 (2)	0.026 (2)	−0.0017 (17)	−0.0053 (17)	−0.0075 (16)
C27A	0.040 (3)	0.036 (2)	0.028 (2)	−0.0003 (18)	−0.0104 (19)	−0.0130 (18)
O1B	0.0386 (17)	0.0279 (15)	0.0329 (16)	−0.0060 (12)	−0.0052 (14)	−0.0094 (12)
O2B	0.0291 (15)	0.0265 (14)	0.0324 (15)	−0.0084 (11)	−0.0100 (12)	−0.0040 (12)
O3B	0.0280 (15)	0.0236 (13)	0.0250 (14)	−0.0037 (11)	−0.0058 (11)	−0.0064 (11)
C1B	0.0171 (19)	0.027 (2)	0.032 (2)	−0.0024 (15)	−0.0081 (16)	−0.0070 (17)
C2B	0.029 (2)	0.029 (2)	0.036 (2)	−0.0018 (17)	−0.0062 (18)	−0.0156 (18)
C3B	0.034 (2)	0.040 (2)	0.026 (2)	0.0029 (18)	−0.0060 (18)	−0.0129 (18)
C4B	0.034 (2)	0.027 (2)	0.030 (2)	−0.0030 (17)	−0.0050 (18)	−0.0033 (17)
C5B	0.025 (2)	0.023 (2)	0.030 (2)	0.0008 (15)	−0.0060 (17)	−0.0105 (16)
C6B	0.0127 (18)	0.0245 (19)	0.032 (2)	0.0008 (14)	−0.0059 (16)	−0.0104 (16)
C7B	0.0121 (18)	0.027 (2)	0.029 (2)	−0.0004 (14)	−0.0098 (15)	−0.0060 (16)
C8B	0.0172 (19)	0.0221 (19)	0.031 (2)	−0.0010 (15)	−0.0056 (16)	−0.0068 (16)
C9B	0.0161 (19)	0.0212 (19)	0.028 (2)	−0.0028 (14)	−0.0046 (15)	−0.0053 (15)
C10B	0.0159 (19)	0.025 (2)	0.030 (2)	−0.0054 (15)	−0.0028 (16)	−0.0078 (16)
C11B	0.0180 (19)	0.030 (2)	0.022 (2)	−0.0036 (15)	−0.0038 (15)	−0.0049 (16)
C12B	0.019 (2)	0.026 (2)	0.033 (2)	−0.0031 (15)	−0.0057 (16)	−0.0059 (17)
C13B	0.0152 (19)	0.027 (2)	0.028 (2)	−0.0052 (15)	−0.0021 (15)	−0.0082 (16)
C14B	0.025 (2)	0.029 (2)	0.021 (2)	−0.0026 (16)	−0.0044 (16)	−0.0067 (16)
C15B	0.023 (2)	0.024 (2)	0.030 (2)	−0.0020 (15)	−0.0055 (17)	−0.0046 (16)
C16B	0.0204 (19)	0.026 (2)	0.024 (2)	−0.0050 (15)	−0.0049 (15)	−0.0031 (16)
C17B	0.0174 (19)	0.028 (2)	0.029 (2)	−0.0025 (15)	−0.0041 (16)	−0.0058 (16)
C18B	0.020 (2)	0.027 (2)	0.026 (2)	−0.0006 (15)	−0.0043 (16)	−0.0061 (16)
C19B	0.022 (2)	0.025 (2)	0.027 (2)	−0.0014 (15)	−0.0054 (16)	−0.0034 (16)
C20B	0.0196 (19)	0.025 (2)	0.024 (2)	−0.0023 (15)	−0.0044 (15)	−0.0046 (15)
C21B	0.019 (2)	0.027 (2)	0.028 (2)	−0.0025 (15)	−0.0023 (16)	−0.0061 (16)
C22B	0.021 (2)	0.028 (2)	0.028 (2)	0.0007 (15)	−0.0052 (16)	−0.0069 (16)
C23B	0.024 (2)	0.025 (2)	0.030 (2)	−0.0040 (16)	−0.0052 (17)	−0.0072 (16)
C24B	0.025 (2)	0.027 (2)	0.028 (2)	−0.0034 (16)	−0.0079 (17)	−0.0068 (16)
C25B	0.027 (2)	0.029 (2)	0.028 (2)	−0.0027 (16)	−0.0070 (17)	−0.0079 (16)
C26B	0.030 (2)	0.031 (2)	0.024 (2)	−0.0031 (17)	−0.0047 (17)	−0.0084 (16)
C27B	0.044 (3)	0.035 (2)	0.033 (2)	−0.0091 (19)	−0.006 (2)	−0.0123 (18)

### Geometric parameters (Å, °)

**Table:**

O1A—C1A	1.353 (4)	O1B—C1B	1.345 (4)
O1A—H1A	0.8200	O1B—H1B	0.8200
O2A—C7A	1.251 (4)	O2B—C7B	1.251 (4)
O3A—C13A	1.366 (4)	O3B—C13B	1.361 (4)
O3A—C16A	1.440 (4)	O3B—C16B	1.440 (4)
C1A—C2A	1.396 (5)	C1B—C2B	1.392 (5)
C1A—C6A	1.407 (5)	C1B—C6B	1.410 (5)
C2A—C3A	1.374 (5)	C2B—C3B	1.375 (5)
C2A—H2AA	0.9300	C2B—H2BA	0.9300
C3A—C4A	1.388 (5)	C3B—C4B	1.392 (5)
C3A—H3AA	0.9300	C3B—H3BA	0.9300
C4A—C5A	1.372 (5)	C4B—C5B	1.366 (5)
C4A—H4AA	0.9300	C4B—H4BA	0.9300
C5A—C6A	1.400 (5)	C5B—C6B	1.403 (5)
C5A—H5AA	0.9300	C5B—H5BA	0.9300
C6A—C7A	1.484 (5)	C6B—C7B	1.473 (5)
C7A—C8A	1.467 (5)	C7B—C8B	1.470 (5)
C8A—C9A	1.337 (5)	C8B—C9B	1.334 (5)
C8A—H8AA	0.9300	C8B—H8BA	0.9300
C9A—C10A	1.459 (5)	C9B—C10B	1.449 (5)
C9A—H9AA	0.9300	C9B—H9BA	0.9300
C10A—C15A	1.403 (5)	C10B—C15B	1.399 (5)
C10A—C11A	1.404 (5)	C10B—C11B	1.399 (5)
C11A—C12A	1.366 (5)	C11B—C12B	1.370 (5)
C11A—H11A	0.9300	C11B—H11B	0.9300
C12A—C13A	1.395 (5)	C12B—C13B	1.397 (5)
C12A—H12A	0.9300	C12B—H12B	0.9300
C13A—C14A	1.387 (5)	C13B—C14B	1.384 (5)
C14A—C15A	1.384 (5)	C14B—C15B	1.389 (5)
C14A—H14A	0.9300	C14B—H14B	0.9300
C15A—H15A	0.9300	C15B—H15B	0.9300
C16A—C17A	1.508 (5)	C16B—C17B	1.507 (5)
C16A—H16A	0.9700	C16B—H16C	0.9700
C16A—H16B	0.9700	C16B—H16D	0.9700
C17A—C18A	1.508 (5)	C17B—C18B	1.517 (5)
C17A—H17A	0.9700	C17B—H17C	0.9700
C17A—H17B	0.9700	C17B—H17D	0.9700
C18A—C19A	1.525 (5)	C18B—C19B	1.522 (5)
C18A—H18A	0.9700	C18B—H18C	0.9700
C18A—H18B	0.9700	C18B—H18D	0.9700
C19A—C20A	1.519 (5)	C19B—C20B	1.511 (5)
C19A—H19A	0.9700	C19B—H19C	0.9700
C19A—H19B	0.9700	C19B—H19D	0.9700
C20A—C21A	1.525 (5)	C20B—C21B	1.515 (5)
C20A—H20A	0.9700	C20B—H20C	0.9700
C20A—H20B	0.9700	C20B—H20D	0.9700
C21A—C22A	1.522 (5)	C21B—C22B	1.521 (5)
C21A—H21A	0.9700	C21B—H21C	0.9700
C21A—H21B	0.9700	C21B—H21D	0.9700
C22A—C23A	1.529 (5)	C22B—C23B	1.518 (5)
C22A—H22A	0.9700	C22B—H22C	0.9700
C22A—H22B	0.9700	C22B—H22D	0.9700
C23A—C24A	1.517 (5)	C23B—C24B	1.519 (5)
C23A—H23A	0.9700	C23B—H23C	0.9700
C23A—H23B	0.9700	C23B—H23D	0.9700
C24A—C25A	1.520 (5)	C24B—C25B	1.531 (5)
C24A—H24A	0.9700	C24B—H24C	0.9700
C24A—H24B	0.9700	C24B—H24D	0.9700
C25A—C26A	1.510 (5)	C25B—C26B	1.519 (5)
C25A—H25A	0.9700	C25B—H25C	0.9700
C25A—H25B	0.9700	C25B—H25D	0.9700
C26A—C27A	1.528 (5)	C26B—C27B	1.523 (5)
C26A—H26A	0.9700	C26B—H26C	0.9700
C26A—H26B	0.9700	C26B—H26D	0.9700
C27A—H27A	0.9600	C27B—H27D	0.9600
C27A—H27B	0.9600	C27B—H27E	0.9600
C27A—H27C	0.9600	C27B—H27F	0.9600
			
C1A—O1A—H1A	109.5	C1B—O1B—H1B	109.5
C13A—O3A—C16A	119.7 (3)	C13B—O3B—C16B	119.3 (3)
O1A—C1A—C2A	117.3 (3)	O1B—C1B—C2B	117.2 (3)
O1A—C1A—C6A	122.7 (3)	O1B—C1B—C6B	122.8 (3)
C2A—C1A—C6A	120.0 (3)	C2B—C1B—C6B	120.0 (3)
C3A—C2A—C1A	119.9 (3)	C3B—C2B—C1B	120.5 (3)
C3A—C2A—H2AA	120.0	C3B—C2B—H2BA	119.7
C1A—C2A—H2AA	120.0	C1B—C2B—H2BA	119.7
C2A—C3A—C4A	120.8 (3)	C2B—C3B—C4B	120.1 (4)
C2A—C3A—H3AA	119.6	C2B—C3B—H3BA	119.9
C4A—C3A—H3AA	119.6	C4B—C3B—H3BA	119.9
C5A—C4A—C3A	119.7 (3)	C5B—C4B—C3B	119.8 (3)
C5A—C4A—H4AA	120.1	C5B—C4B—H4BA	120.1
C3A—C4A—H4AA	120.1	C3B—C4B—H4BA	120.1
C4A—C5A—C6A	121.1 (3)	C4B—C5B—C6B	121.7 (3)
C4A—C5A—H5AA	119.5	C4B—C5B—H5BA	119.2
C6A—C5A—H5AA	119.5	C6B—C5B—H5BA	119.2
C5A—C6A—C1A	118.5 (3)	C5B—C6B—C1B	117.8 (3)
C5A—C6A—C7A	122.4 (3)	C5B—C6B—C7B	123.1 (3)
C1A—C6A—C7A	119.1 (3)	C1B—C6B—C7B	119.1 (3)
O2A—C7A—C8A	119.3 (3)	O2B—C7B—C8B	119.0 (3)
O2A—C7A—C6A	118.8 (3)	O2B—C7B—C6B	119.8 (3)
C8A—C7A—C6A	121.9 (3)	C8B—C7B—C6B	121.2 (3)
C9A—C8A—C7A	119.6 (3)	C9B—C8B—C7B	120.5 (3)
C9A—C8A—H8AA	120.2	C9B—C8B—H8BA	119.7
C7A—C8A—H8AA	120.2	C7B—C8B—H8BA	119.7
C8A—C9A—C10A	129.0 (3)	C8B—C9B—C10B	128.6 (3)
C8A—C9A—H9AA	115.5	C8B—C9B—H9BA	115.7
C10A—C9A—H9AA	115.5	C10B—C9B—H9BA	115.7
C15A—C10A—C11A	117.7 (3)	C15B—C10B—C11B	117.3 (3)
C15A—C10A—C9A	119.3 (3)	C15B—C10B—C9B	119.7 (3)
C11A—C10A—C9A	123.0 (3)	C11B—C10B—C9B	123.0 (3)
C12A—C11A—C10A	120.7 (3)	C12B—C11B—C10B	121.3 (3)
C12A—C11A—H11A	119.6	C12B—C11B—H11B	119.4
C10A—C11A—H11A	119.6	C10B—C11B—H11B	119.4
C11A—C12A—C13A	121.0 (3)	C11B—C12B—C13B	120.5 (3)
C11A—C12A—H12A	119.5	C11B—C12B—H12B	119.8
C13A—C12A—H12A	119.5	C13B—C12B—H12B	119.8
O3A—C13A—C14A	124.9 (3)	O3B—C13B—C14B	125.2 (3)
O3A—C13A—C12A	115.5 (3)	O3B—C13B—C12B	115.0 (3)
C14A—C13A—C12A	119.6 (3)	C14B—C13B—C12B	119.8 (3)
C15A—C14A—C13A	119.4 (3)	C13B—C14B—C15B	119.0 (3)
C15A—C14A—H14A	120.3	C13B—C14B—H14B	120.5
C13A—C14A—H14A	120.3	C15B—C14B—H14B	120.5
C14A—C15A—C10A	121.7 (3)	C14B—C15B—C10B	122.2 (3)
C14A—C15A—H15A	119.1	C14B—C15B—H15B	118.9
C10A—C15A—H15A	119.1	C10B—C15B—H15B	118.9
O3A—C16A—C17A	106.4 (3)	O3B—C16B—C17B	106.2 (3)
O3A—C16A—H16A	110.4	O3B—C16B—H16C	110.5
C17A—C16A—H16A	110.4	C17B—C16B—H16C	110.5
O3A—C16A—H16B	110.4	O3B—C16B—H16D	110.5
C17A—C16A—H16B	110.4	C17B—C16B—H16D	110.5
H16A—C16A—H16B	108.6	H16C—C16B—H16D	108.7
C16A—C17A—C18A	115.6 (3)	C16B—C17B—C18B	114.6 (3)
C16A—C17A—H17A	108.4	C16B—C17B—H17C	108.6
C18A—C17A—H17A	108.4	C18B—C17B—H17C	108.6
C16A—C17A—H17B	108.4	C16B—C17B—H17D	108.6
C18A—C17A—H17B	108.4	C18B—C17B—H17D	108.6
H17A—C17A—H17B	107.4	H17C—C17B—H17D	107.6
C17A—C18A—C19A	111.6 (3)	C17B—C18B—C19B	111.2 (3)
C17A—C18A—H18A	109.3	C17B—C18B—H18C	109.4
C19A—C18A—H18A	109.3	C19B—C18B—H18C	109.4
C17A—C18A—H18B	109.3	C17B—C18B—H18D	109.4
C19A—C18A—H18B	109.3	C19B—C18B—H18D	109.4
H18A—C18A—H18B	108.0	H18C—C18B—H18D	108.0
C20A—C19A—C18A	116.1 (3)	C20B—C19B—C18B	116.2 (3)
C20A—C19A—H19A	108.3	C20B—C19B—H19C	108.2
C18A—C19A—H19A	108.3	C18B—C19B—H19C	108.2
C20A—C19A—H19B	108.3	C20B—C19B—H19D	108.2
C18A—C19A—H19B	108.3	C18B—C19B—H19D	108.2
H19A—C19A—H19B	107.4	H19C—C19B—H19D	107.4
C19A—C20A—C21A	112.7 (3)	C19B—C20B—C21B	112.7 (3)
C19A—C20A—H20A	109.0	C19B—C20B—H20C	109.1
C21A—C20A—H20A	109.0	C21B—C20B—H20C	109.1
C19A—C20A—H20B	109.0	C19B—C20B—H20D	109.1
C21A—C20A—H20B	109.0	C21B—C20B—H20D	109.1
H20A—C20A—H20B	107.8	H20C—C20B—H20D	107.8
C22A—C21A—C20A	114.4 (3)	C20B—C21B—C22B	115.0 (3)
C22A—C21A—H21A	108.6	C20B—C21B—H21C	108.5
C20A—C21A—H21A	108.6	C22B—C21B—H21C	108.5
C22A—C21A—H21B	108.6	C20B—C21B—H21D	108.5
C20A—C21A—H21B	108.6	C22B—C21B—H21D	108.5
H21A—C21A—H21B	107.6	H21C—C21B—H21D	107.5
C21A—C22A—C23A	113.3 (3)	C23B—C22B—C21B	113.7 (3)
C21A—C22A—H22A	108.9	C23B—C22B—H22C	108.8
C23A—C22A—H22A	108.9	C21B—C22B—H22C	108.8
C21A—C22A—H22B	108.9	C23B—C22B—H22D	108.8
C23A—C22A—H22B	108.9	C21B—C22B—H22D	108.8
H22A—C22A—H22B	107.7	H22C—C22B—H22D	107.7
C24A—C23A—C22A	113.7 (3)	C22B—C23B—C24B	114.0 (3)
C24A—C23A—H23A	108.8	C22B—C23B—H23C	108.7
C22A—C23A—H23A	108.8	C24B—C23B—H23C	108.7
C24A—C23A—H23B	108.8	C22B—C23B—H23D	108.7
C22A—C23A—H23B	108.8	C24B—C23B—H23D	108.7
H23A—C23A—H23B	107.7	H23C—C23B—H23D	107.6
C23A—C24A—C25A	113.9 (3)	C23B—C24B—C25B	114.2 (3)
C23A—C24A—H24A	108.8	C23B—C24B—H24C	108.7
C25A—C24A—H24A	108.8	C25B—C24B—H24C	108.7
C23A—C24A—H24B	108.8	C23B—C24B—H24D	108.7
C25A—C24A—H24B	108.8	C25B—C24B—H24D	108.7
H24A—C24A—H24B	107.7	H24C—C24B—H24D	107.6
C26A—C25A—C24A	113.7 (3)	C26B—C25B—C24B	113.7 (3)
C26A—C25A—H25A	108.8	C26B—C25B—H25C	108.8
C24A—C25A—H25A	108.8	C24B—C25B—H25C	108.8
C26A—C25A—H25B	108.8	C26B—C25B—H25D	108.8
C24A—C25A—H25B	108.8	C24B—C25B—H25D	108.8
H25A—C25A—H25B	107.7	H25C—C25B—H25D	107.7
C25A—C26A—C27A	113.5 (3)	C25B—C26B—C27B	113.4 (3)
C25A—C26A—H26A	108.9	C25B—C26B—H26C	108.9
C27A—C26A—H26A	108.9	C27B—C26B—H26C	108.9
C25A—C26A—H26B	108.9	C25B—C26B—H26D	108.9
C27A—C26A—H26B	108.9	C27B—C26B—H26D	108.9
H26A—C26A—H26B	107.7	H26C—C26B—H26D	107.7
C26A—C27A—H27A	109.5	C26B—C27B—H27D	109.5
C26A—C27A—H27B	109.5	C26B—C27B—H27E	109.5
H27A—C27A—H27B	109.5	H27D—C27B—H27E	109.5
C26A—C27A—H27C	109.5	C26B—C27B—H27F	109.5
H27A—C27A—H27C	109.5	H27D—C27B—H27F	109.5
H27B—C27A—H27C	109.5	H27E—C27B—H27F	109.5
			
O1A—C1A—C2A—C3A	−179.1 (3)	O1B—C1B—C2B—C3B	−179.6 (3)
C6A—C1A—C2A—C3A	0.6 (5)	C6B—C1B—C2B—C3B	−0.7 (5)
C1A—C2A—C3A—C4A	0.8 (6)	C1B—C2B—C3B—C4B	0.7 (6)
C2A—C3A—C4A—C5A	−1.2 (6)	C2B—C3B—C4B—C5B	−0.1 (6)
C3A—C4A—C5A—C6A	0.2 (6)	C3B—C4B—C5B—C6B	−0.6 (6)
C4A—C5A—C6A—C1A	1.2 (5)	C4B—C5B—C6B—C1B	0.6 (5)
C4A—C5A—C6A—C7A	−177.9 (3)	C4B—C5B—C6B—C7B	−179.4 (3)
O1A—C1A—C6A—C5A	178.1 (3)	O1B—C1B—C6B—C5B	178.9 (3)
C2A—C1A—C6A—C5A	−1.5 (5)	C2B—C1B—C6B—C5B	0.1 (5)
O1A—C1A—C6A—C7A	−2.8 (5)	O1B—C1B—C6B—C7B	−1.1 (5)
C2A—C1A—C6A—C7A	177.6 (3)	C2B—C1B—C6B—C7B	180.0 (3)
C5A—C6A—C7A—O2A	178.4 (3)	C5B—C6B—C7B—O2B	175.9 (3)
C1A—C6A—C7A—O2A	−0.7 (5)	C1B—C6B—C7B—O2B	−4.1 (5)
C5A—C6A—C7A—C8A	−2.7 (5)	C5B—C6B—C7B—C8B	−5.4 (5)
C1A—C6A—C7A—C8A	178.2 (3)	C1B—C6B—C7B—C8B	174.7 (3)
O2A—C7A—C8A—C9A	6.5 (5)	O2B—C7B—C8B—C9B	8.8 (5)
C6A—C7A—C8A—C9A	−172.3 (3)	C6B—C7B—C8B—C9B	−169.9 (3)
C7A—C8A—C9A—C10A	179.9 (3)	C7B—C8B—C9B—C10B	−179.7 (3)
C8A—C9A—C10A—C15A	−168.6 (4)	C8B—C9B—C10B—C15B	−169.5 (4)
C8A—C9A—C10A—C11A	13.0 (6)	C8B—C9B—C10B—C11B	10.8 (6)
C15A—C10A—C11A—C12A	0.1 (5)	C15B—C10B—C11B—C12B	−0.2 (5)
C9A—C10A—C11A—C12A	178.5 (3)	C9B—C10B—C11B—C12B	179.5 (3)
C10A—C11A—C12A—C13A	0.2 (5)	C10B—C11B—C12B—C13B	0.3 (5)
C16A—O3A—C13A—C14A	−6.8 (5)	C16B—O3B—C13B—C14B	−12.2 (5)
C16A—O3A—C13A—C12A	173.7 (3)	C16B—O3B—C13B—C12B	168.4 (3)
C11A—C12A—C13A—O3A	179.8 (3)	C11B—C12B—C13B—O3B	178.9 (3)
C11A—C12A—C13A—C14A	0.3 (5)	C11B—C12B—C13B—C14B	−0.5 (5)
O3A—C13A—C14A—C15A	179.5 (3)	O3B—C13B—C14B—C15B	−178.6 (3)
C12A—C13A—C14A—C15A	−1.0 (5)	C12B—C13B—C14B—C15B	0.8 (5)
C13A—C14A—C15A—C10A	1.4 (5)	C13B—C14B—C15B—C10B	−0.7 (5)
C11A—C10A—C15A—C14A	−0.9 (5)	C11B—C10B—C15B—C14B	0.5 (5)
C9A—C10A—C15A—C14A	−179.4 (3)	C9B—C10B—C15B—C14B	−179.2 (3)
C13A—O3A—C16A—C17A	175.0 (3)	C13B—O3B—C16B—C17B	177.5 (3)
O3A—C16A—C17A—C18A	176.8 (3)	O3B—C16B—C17B—C18B	176.5 (3)
C16A—C17A—C18A—C19A	−176.9 (3)	C16B—C17B—C18B—C19B	−178.8 (3)
C17A—C18A—C19A—C20A	−176.2 (3)	C17B—C18B—C19B—C20B	−173.5 (3)
C18A—C19A—C20A—C21A	−179.7 (3)	C18B—C19B—C20B—C21B	−176.4 (3)
C19A—C20A—C21A—C22A	−174.4 (3)	C19B—C20B—C21B—C22B	−174.4 (3)
C20A—C21A—C22A—C23A	175.3 (3)	C20B—C21B—C22B—C23B	177.0 (3)
C21A—C22A—C23A—C24A	−177.5 (3)	C21B—C22B—C23B—C24B	−178.3 (3)
C22A—C23A—C24A—C25A	176.3 (3)	C22B—C23B—C24B—C25B	173.9 (3)
C23A—C24A—C25A—C26A	179.6 (3)	C23B—C24B—C25B—C26B	178.6 (3)
C24A—C25A—C26A—C27A	−179.5 (3)	C24B—C25B—C26B—C27B	−174.1 (3)

### Hydrogen-bond geometry (Å, °)

**Table:**

*D*—H···*A*	*D*—H	H···*A*	*D*···*A*	*D*—H···*A*
O1A—H1A···O2A	0.82	1.79	2.513 (4)	146
O1B—H1B···O2B	0.82	1.81	2.530 (4)	146
C22B—H22C···Cg1^i^	0.97	2.77	3.654 (4)	151
C16B—H16D···Cg2^i^	0.97	3.00	3.736 (4)	134
C17B—H17D···Cg2	0.97	2.82	3.612 (4)	139
C22A—H22B···Cg3	0.97	2.93	3.765 (4)	145

Symmetry codes: (i) *x*−1, *y*, *z*.
